# Maternal Lecithin Supplementation in Sows Regulates the Hepatic Glycolipid Metabolism of Offspring

**DOI:** 10.3390/ani15182685

**Published:** 2025-09-13

**Authors:** Xudong Yang, Haoyang Wang, Juan Xiong, Chunyan Xie, Hongjun Yang, Liuan Li

**Affiliations:** 1 Tianjin Key Laboratory of Agricultural Animal Breeding and Healthy Husbandry, College of Animal Science and Veterinary Medicine, Tianjin Agricultural University, Tianjin 300392, China; yangxudong2022@126.com (X.Y.); roronoaedward@outlook.com (H.W.); 2 Shanchuan Bioechnology Co., Ltd., Wuhan 430070, China; xiongjuan@anschina.cn (J.X.); yanghongjun@anschina.cn (H.Y.); 3 Tianjin Key Laboratory of Animal Molecular Breeding and Biotechnology, Tianjin Livestock and Poultry Health Breeding Technology Engineering Center, Institute of Animal Science and Veterinary, Tianjin Academy of Agricultural Sciences, Tianjin 300381, China

**Keywords:** lecithin, phospholipid, glucose, hepatic metabolism, sow, suckling piglets

## Abstract

Maternal nutrition during gestation and lactation influences offspring development and metabolic health. Lecithin, a crucial phospholipid commonly used in animal diets to improve lipid absorption and energy metabolism, is a viable approach to optimize this synergy and improve neonatal resilience. This study aimed to investigate the regulation mechanism of maternal lecithin supplementation during late gestation and lactation on the growth performance and hepatic glycolipid metabolism of offspring. Twenty-four multiparous sows were randomly allocated to receive either a control (CON) diet or a lecithin-supplemented diet (2 kg/t lecithin) on day 95 of gestation until weaning (day 21 postpartum). Blood samples were collected from sows during late gestation and on day 21 of lactation, as well as from umbilical cords and weaned piglets at 21 days. Results showed that suckling piglets from lecithin group had faster growth rate, especially during 1–7 d of suckling period. Lecithin supplement reduced the serum cholesterol of pregnant sows, while not affected it in serum of umbilical cord. In contrast, both the serum cholesterol and glucose level presented upward trend in lactating sows in the lecithin group. Accordingly, maternal lecithin supplement increased the serum glucose level, as well as the levels of hepatic lipid and glucose levels, accompanied by decreasing the hepatic levels of certain long-chain fatty acids of 21-day-old piglets compared with the CON group. Furthermore, maternal lecithin supplement activated hepatic glycolipid metabolism via regulating the mRNA level of hepatic gluconeogenesis and lipid synthesis in 21-day-old piglets. In short, this study demonstrated that maternal lecithin supplementation improved hepatic energy homeostasis in offspring by activating gluconeogenesis and optimizing lipid storage, thereby enhancing neonatal growth performance and metabolic resilience for weaning challenges.

## Simple Summary

Mother’s nutrition during pregnancy and lactation is important for piglet growth. In this study, sows were treated with lecithin-containing feed from the 95th day of gestation to weaning (21 days postpartum) to explore the effects of lecithin supplementation, a phospholipid that enhances fat emulsification and energy utilization, on the growth of young piglets. The results showed that young pigs supplemented with lecithin grew faster, had higher serum and liver glucose levels, and reduced certain long-chain fatty acids in the liver during the early lactation period. This may be because lecithin activates gluconeogenesis in the liver of young pigs, optimizing lipid storage. This research will help improve sow nutrition, improve young pig growth performance and cope with weaning, and have practical value for the breeding industry.

## 1. Introduction

Nutritional supplementation during gestation and lactation in sows is essential for the survival of newborns, the lactation process, and the growth of piglets [1]. Early weaning improves sow reproductive efficiency [2]; however, it negatively affects piglet nutrient utilization and leads to detrimental metabolic outcomes. Newborns depend exclusively on endogenous reserves for glycemic control prior to colostrum intake, rendering these reserves essential for survival [3]. During weaning, the immature digestive systems of piglets must adapt concurrently to unfamiliar eating patterns and increased metabolic demands [4]. These challenges are exacerbated by impaired hepatic glucose–lipid metabolism [5,6,7], requiring piglets to adapt through enhanced gluconeogenesis and modified lipid metabolism. Optimizing nutrient efficiency and energy metabolism in piglets enhances metabolic adaptation and growth performance. This adaptation is based on the nutritional synergy between mother and offspring, in which maternal nutrition during gestation and lactation influences the long-term metabolic capacity of the offspring [8]. The placenta, as a nutrient-sensing interface, mediates this synergy by converting maternal signals into epigenetic modifications of fetal hepatic genes [9,10]. This nutrient continuity is essential in influencing the offspring’s capacity to sustain glucose–lipid homeostasis during later challenges such as weaning [11,12].

Lecithin, a key phospholipid derived from soybeans, serves as a multifunctional nutrient, building upon the nutritional synergy between mother and offspring [13,14]. It improves cell membrane fluidity, facilitating embryonic development [15], as evidenced by enhanced hatchability and embryonic nutrition in poultry [16]. Lecithin supplementation in sows during late gestation significantly increases weaning litter weight, reduces backfat loss, decreases the incidence of intrauterine growth restriction (IUGR), and improves piglet birth outcomes. Simultaneously, it enhances maternal energy utilization and decreases body fat mobilization in sows [13,14,17]. It promotes lipid metabolism and reduces hepatic fat deposition, thereby optimizing energy availability [17,18], indicating a systematic metabolic regulatory effect.

The mechanism by which maternal lecithin influences offspring metabolic programming remains poorly understood, particularly in terms of regulating hepatic glucose and lipid metabolism across generations. Lecithin serves a dual role as both a component of membrane structure and a metabolic regulator. We hypothesize that it optimizes the mother–offspring metabolic axis by programming hepatic energy homeostasis in piglets. To confirm this, we supplemented maternal diets with lecithin during late pregnancy and lactation to assess its effects on the growth and hepatic glucose and lipid metabolism of suckling piglets, as well as to elucidate the regulatory mechanisms underlying the metabolic adaptation of the offspring’s liver. We will examine the function of lecithin in facilitating a coordinated energy distribution system between the mother and offspring, as well as its role in improving the adaptability of piglets during the weaning transition period.

## 2. Materials and Methods

### 2.1. Experimental Design

A total of 24 multiparous sows (Landrace × Yorkshire; parity 3–5) with a uniform genetic background, similar body condition (backfat thickness: 18–22 mm), and expected farrowing date were selected and randomly allocated to two dietary treatments: (1) CON (basal diet) and (2) Lecithin (basal diet supplemented with 2 kg/t lecithin). The lecithin dosage was established via preliminary trials and procured commercially from Shanchuan Biotechnology Co., Ltd. (Wuhan, China).

The lecithin supplementation level (2 kg/t diet) was determined based on previous studies demonstrating its efficacy in improving reproductive performance and lipid metabolism in sows [17] and was validated in our preliminary trials. Sows were individually housed and fed. The daily feed allowance was 2.0–2.4 g twice daily during late gestation (totaling 4.0–4.8 g/d) and increased to 8 g/d from day 3 of lactation. Accordingly, the daily lecithin intake was calculated to be 8.0–9.6 g/sow during late gestation and 16 g/sow during peak lactation. The experimental protocol commenced on day 95 of gestation and persisted throughout the lactation period until weaning at 21 days postpartum.

The diets were selected as our previous described which formulated to satisfy the nutritional standards established by the NRC (2012) [19] and could meet the nutritional requirements of sows during gestation and lactation (Table 1). All experimental animals were housed in individual slatted-floor pens with unrestricted access to water. In the postpartum phase, sows were fed at 06:30 and 16:30 daily, adhering to a quantitatively regulated feeding protocol. The initial provision was 2.0–2.4 g per feeding, with a progressive increment, transitioning to standardized rations of 8 g/d from lactation day 3 onward, in accordance with the operational protocols of Yangxinjiahe Modern Agriculture Co., Ltd. (Huangshi, China), where the trial was conducted.

### 2.2. Sample Collection

Parturition parameters, such as total born, liveborn, stillborn, incidence of intrauterine growth restriction (IUGR), and neonatal birth weights, were systematically documented for each sow on the day of delivery [20]. Six sows from each group were subjected to biological sampling during the peripartum period. Approximately 5 mL of blood was obtained from the ear vein of the sows on day 110 of gestation, at farrowing, and on day 21 of lactation. At farrowing, 5 mL umbilical cord blood samples were collected from the same sows. The samples were centrifuged at 3000× *g* at 4 °C for 10 min, after which the serum was aliquoted and stored at −80 °C for subsequent analysis [21].

At weaning (day 21 postpartum), one piglet per litter (from the six sows per group selected for blood sampling) was chosen based on representing the average body weight of the litter to avoid outliers. Only male piglets were included. After blood collection (5 mL via cardiac puncture following anesthesia with sodium pentobarbital administered via IV injection at 50 mg/kg BW), piglets were euthanized by exsanguination [22]. Liver samples were collected: one portion was snap-frozen in liquid nitrogen and stored at −80 °C for RNA extraction and q-PCR analysis, while another portion was stored at −20 °C for subsequent analysis of lipid and metabolite content.

### 2.3. Sample Analysis

#### 2.3.1. Serum Biochemical Profile

Serum biochemical profiles were conducted using spectrophotometry with commercial enzymatic assay kits (Roche Diagnostics, Montreal, QC, Canada), on a Beckman Coulter Synchron CX Pro clinical chemistry analyzer (Beckman Coulter, CA, USA). Indicators including triglycerides (TG), cholesterol (TC), low-density lipoprotein cholesterol (LDL), high-density lipoprotein cholesterol (HDL), glucose (GLU), D-lactate (LACT), cholinesterase (CHE), lactate dehydrogenase (LDHI), and lipase (LIPC) were detected, with all procedures conducted in accordance with the manufacturer’s standardized protocols.

#### 2.3.2. Hepatic GLU, Glycine, TG and TC Contens

Parameters of hepatic lipid metabolism TG and TC were measured using enzymatic colorimetry with tissue-specific assay kits (E1013/E1015, Beijing Pulai Gene Technology, Beijing, China). Carbohydrate metabolic indices GLU and GLY were assessed using biochemical detection systems (ZC-SO418/SO420, Shanghai Zhuocai Biotechnology, Shanghai, China). All analytical procedures were conducted in accordance with the protocols specified by the manufacturer for processing tissue homogenates.

#### 2.3.3. Serum and Hepatic Medium and Long-Chain Fatty Acids Proportion

For fatty acids (FA’s) proportion analysis, 1 mL serum sample was extracted overnight at 50 °C in a water bath with 5% acetyl chloride/methanol solution. And then, 1 mL of n-hexane was added and centrifuged at 2500× *g* at 4 °C for 5 min. Finally, the supernatant was collected and used to determine FA’s composition. In addition, the FA’s in the liver of piglets was extracted with chloroform-methanolic KOH. The gas chromatography (Agilent 6890, Boston, MA, USA) was used to analyze the FA’s profiles, and the results were expressed as a percentage of total FA’s.

#### 2.3.4. Quantitative Real-Time PCR (qRT-PCR)

Liver tissues were homogenized using liquid nitrogen, and messenger RNA (mRNA) was extracted according to established protocols [23]. Transcript quantification of glycolipid metabolic targets was conducted using quantitative reverse transcription PCR (qRT-PCR) with Thermo Scientific Luminaris Color HiGreen High ROX master mix (Waltham, MA, USA) on a Bio-Rad iCycler thermal cycler (Hercules, CA, USA), utilizing constitutively expressed GAPDH as the endogenous control. Custom-synthesized oligonucleotide primers (Table 2) facilitated specific amplification, with relative expression dynamics determined using the comparative threshold cycle (2^−ΔΔCT^) algorithm, in accordance with MIQE-compliant experimental protocols.

#### 2.3.5. Statistical Analysis

Data were analyzed using SPSS Statistics 21.0 (IBM Corp., Armonk, NY, USA). The sow was considered the experimental unit for sow performance and serum parameters. The piglet was considered the experimental unit for offspring data, data are presented as mean ± SEM. For the main analysis of treatment effects, a two-tailed Student’s T-test was used with probability thresholds established as follows: *p* < 0.05 (95% confidence interval) for statistical significance and 0.05 < *p* < 0.10 considered suggestive of a statistical trend. Data visualization was conducted using GraphPad Prism 7.00 (La Jolla, CA, USA) graphical software.

## 3. Results

### 3.1. Productive Performance of Sows

The production performance of sows is presented in Table 3 and Table 4. No significant differences (*p* > 0.10) were observed between groups for total born, born alive, stillbirth, or IUGR incidence. Maternal lecithin supplementation tends to enhance the daily weight gain of suckling piglets from 1 to 7 days (*p* = 0.08). Adding lecithin to the diet did not significantly impact the reproductive performance of sows (*p* > 0.10).

### 3.2. Serum Biochemical Parameters of Sow at Late Pregnancy and Lactating

The serum biochemical parameters of gestating sows are detailed in Table 5. In late gestation, dietary lecithin supplementation markedly decreased serum TC and high-density lipoprotein (HDL) levels relative to the control group (*p* < 0.05). During lactation, sows demonstrated significantly higher serum cholinesterase (CHE) and total bile acid (TBA) concentrations (*p* < 0.05), as well as a trend towards increased total cholesterol (TC), low-density lipoprotein (LDL), high-density lipoprotein (HDL), and glucose (GLU) levels (*p* < 0.10) following lecithin treatment.

### 3.3. Serum Biochemical Parameters of Umbilical Cord and Suckling Piglets at 21 d

Serum biochemical parameters of umbilical cord blood of sows and 21-day-old weaned piglets were summarized as shown in Table 6. Dietary lecithin supplementation significantly elevated hepatic lipase (LIPC) levels in the umbilical cord serum of sows compared to the control group (*p* < 0.05). Maternal Lecithin supplementation significantly increased serum glucose (GLU) concentration in suckling piglets (*p* < 0.05) relative to the control group.

### 3.4. Serum and Hepatic Fatty Acid Profiles in Sow and Suckling Piglets

The effects of maternal dietary lecithin on serum and liver fatty acids in piglets during a 21-day lactation period are summarized in Table 7. Maternal dietary Lecithin supplementation significantly reduced C18:1n9t, C20:3n6, and C24:0 levels in the liver of piglets at 21 days of lactation when compared to the CON group (*p* < 0.05). A tendency for an increase in C20:0 levels was observed in the liver of piglets at 21 days of lactation (*p* < 0.10).

### 3.5. Hepatic TC, TG, GLU, GLY Contents and the Related Metabolic Gene Expression in Weaned Piglets

**Figure 1:**
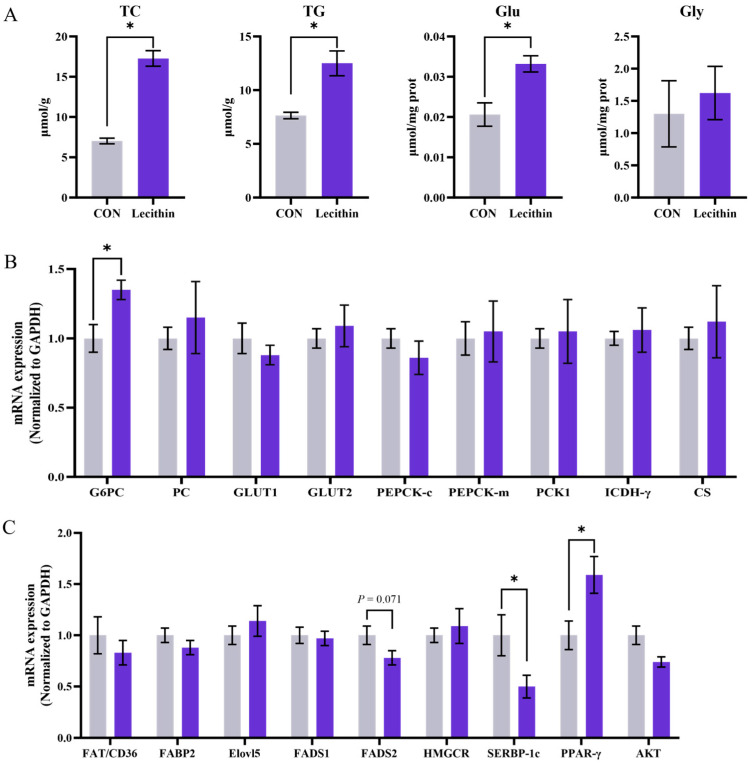
The effect of dietary lecithin supplementation in sows on the liver of piglets at 21 d of lactation. (**A**) TC, TG, Glu and Gly in the liver of piglets. (**B**) mRNA expression of glucose metabolism-related genes in the liver of piglets. (**C**) mRNA expression of lipid metabolism-related genes in the liver of piglets. TC: cholesterol, TG: triglyceride, Glu: glucose, Gly: glycogen. G6PC: glucose-6-phosphatase catalytic subunit; GLUT1: glucose transporter-1; GLUT2: glucose transporter-2; PEPCK-c: phosphoenolpyruvate carboxykinase-1; PEPCK-m: phosphoenolpyruvate carboxykinase-2; PCK1: Phosphoenolpyruvate carboxykinase 1; ICDH-γ: Isocitrate dehydrogenase-γ; CS: Citrate synthase; FAT/CD36: fatty acid transporter/CD36; FABP2: fatty acid binding protein 2; ELOVL5: elongation of very long chain fatty acids protein 5; FADS1: fatty acid desaturase 1; FADS2: fatty acid desaturase 2; HMGCR: hmgcr-3-hydroxy-3-methylglutaryl-CoA reductase; SREBP-1c: sterol-regulatory element binding proteins; PPARγ: peroxisome proliferator-activated receptor-γ; AKT: protein kinase B; GAPDH: glyceraldehyde-3-phosphate dehydrogenase; Data is presented as mean ± SEM, n = 6. Statistical significance was set at * *p* < 0.05.

The hepatic levels of triglycerides (TG), total cholesterol (TC), glucose (Glu), and glycogen (Gly) in piglets are illustrated in Figure 1. Maternal dietary Lecithin supplementation significantly elevated TC, TG, and GLU levels in the liver of piglets at 21 days of lactation compared to the CON group (*p* < 0.05) (Figure 1A). In addition, relative to the CON group, the expression levels of Glucose-6-phosphatase catalytic subunit (G6PC) and peroxisome proliferator-activated receptor-γ (PPAR-γ) were significantly elevated in the liver of suckling piglets from the Lecithin group (*p* < 0.05) (Figure 1B). Furthermore, in comparison to the CON group, the levels of sterol-regulatory element binding proteins (SERBP-1c) were significantly reduced in the liver of suckling piglets from the Lecithin group (*p* < 0.05). Additionally, there was a trend towards a decrease in the expression of fatty acid desaturase 2 (FADS2) in the liver of piglets at 21 days of lactation (*p* < 0.10) (Figure 1C).

## 4. Discussion

The growth performance of piglets is crucial for the economic viability of pig production [24]. This study found that piglets supplemented with maternal lecithin supplementation showed a tendency for increased average daily gain (ADG) from day 1 to 7, consistent with previous findings by Saseendran et al. on the growth-promoting effects of lecithin [25]. Neonatal piglets rely heavily on endogenous energy reserves to manage parturition stress and facilitate rapid early growth [3,26]. Significantly elevated serum GLU and hepatic GLU levels were observed in the piglets of treatment groups, suggesting that lecithin is of considerable physiological importance in augmentation of glycogen reserves in offspring. Maternal lecithin supplementation enhances the energy metabolism of lactating sows, as indicated by increased levels of GLU and TC. Conversely, it modulates the hepatic glucose metabolism pathway in piglets. G6PC, as a rate-limiting enzyme in gluconeogenesis [27], regulates glucose release through the hydrolysis of glucose-6-phosphate. Lecithin-induced upregulation of G6PC contributes to enhanced hepatic glucose production and likely underlies the elevated hepatic and circulating glucose levels observed. This suggests that lecithin activates gluconeogenesis, enabling piglets to maintain glucose homeostasis under fluctuating nutrient conditions. The improvement in gluconeogenic capacity is essential for sustaining blood glucose homeostasis during the post-weaning phase characterized by variable food intake. In the absence of exogenous nutrient supply, piglets can utilize the activated hepatic gluconeogenesis pathway to stabilize blood glucose levels and ensure a continuous energy supply to essential organs, including the brain [5].

Piglet liver directly or indirectly supports its growth and development by providing energy and regulating intestinal health through lipid metabolism [28]. This experiment demonstrates that lecithin, characterized by its hydrophilic head and hydrophobic tail, can effectively emulsify fat particles. This process enhances their susceptibility to enzymatic hydrolysis and transportation, facilitating intestinal absorption and decreasing the liver’s requirement to synthesize endogenous fat [29,30]. Consequently, the decreased hepatic TG and TC levels in piglets born to sows supplemented with lecithin demonstrates that the lipids are more efficiently transported from the liver to peripheral tissues, thereby preventing hepatic accumulation in piglets. Similar to this characterization were the results at the genes level in the liver of reduced expression of SREBP-1c, as a key transcription factor regulating the de novo synthesis of fatty acids and triglycerides [31], indicating that maternal lecithin may inhibit the synthetic pathways of endogenous hepatic fatty acids and triglycerides in piglets [32]. This finding aligns with earlier studies indicating that lecithin may decrease liver fat accumulation in various species through comparable mechanisms [17]. Interestingly, the expression of PPARγ-a key regulator of lipid uptake and storage-was upregulated. This regulation, despite appearing contradictory, serves as an effective strategy for energy allocation. Piglets can improve energy efficiency by increasing the absorption and storage of external lipids, such as those derived from breast milk, while decreasing the energy-intensive process of endogenous lipid synthesis [33,34]. Moreover, lecithin supplementation significantly reduced the proportions of C18:1n9t, C20:3n6, and C24:0 in piglet livers, which was associated with decreased FADS2 expression. FADS2, the rate-limiting enzyme in PUFA synthesis [35], exhibits downregulation that correlates with a significant decrease in hepatic C20:3n6 levels. Previous studies have shown that phospholipids can influence hepatic lipoprotein metabolism and create a regulatory network involving lecithin, FADS2, and fatty acid metabolism, indicating that lecithin affects fatty acid desaturation in piglet livers. The overall proportions of SFA, MUFA, and PUFA remained unchanged; however, the modifications in the fatty acid profile may have unique physiological implications, necessitating further experimental validation of the specific mechanisms involved.

This research noted substantial alterations in the metabolic status of sows resulting from lecithin supplementation. In late gestation, dietary lecithin markedly decreased serum total cholesterol and HDL levels in sows, indicating improved cholesterol utilization for fetal development or hormone synthesis. During the lactation period, significant increases in CHE and total bile acid (TBA) concentrations were observed, alongside rising trends in serum TC, LDL, HDL, GLU, and hepatic LIPC levels. The metabolic alterations indicate that maternal lecithin supplementation significantly enhances lipid metabolism in lactating sows via various pathways. CHE is involved in lipoprotein metabolism; elevated TBA signifies increased bile acid synthesis and lipid digestion capacity, whereas LIPC facilitates triglyceride hydrolysis in lipoproteins [36,37,38]. The coordinated changes collectively improve the sow’s capacity to mobilize, transport, digest, and utilize lipids. This metabolic adaptation fulfills two physiological functions: This approach addresses significant energy requirements during high-yield lactation while reducing maternal backfat loss [39,40]. Secondly, it likely optimizes the composition of milk, including energy density, phospholipid content, and fatty acid profile, thereby indirectly affecting piglet metabolism. The metabolic changes observed in sows seem to interact with the hepatic metabolic adaptations in piglets, creating a cohesive mother–offspring metabolic axis that supports neonatal growth and development. The failure to collect colostrum samples from the sows in a timely manner constitutes a limitation of the current study, as it results in missing data on sow colostrum composition, while it could provide evidence for the application of lecithin on sows and thus regulation of offspring metabolism. The interdependent changes form a cohesive maternal–offspring metabolic axis. The incorporation of lecithin enhances maternal nutrient utilization and optimizes the energy storage efficiency of offspring, thereby reducing maternal reserve depletion and increasing the readiness of neonates for weaning.

## 5. Conclusions

This study demonstrates that maternal lecithin supplementation during late gestation and lactation improves hepatic glucose and lipid metabolism in suckling piglets. This may be achieved by activating G6PC-mediated gluconeogenesis and optimizing PPARγ/SREBP-1c-regulated lipid storage within the liver. Concurrently, lecithin enhances lipid mobilization efficiency in lactating sows. Together, these maternal and offspring metabolic adaptations improve neonatal growth performance and prepare piglets for the metabolic challenges of weaning.

## Figures and Tables

**Table 1:** Ingredients and nutrient levels of the basal diet for late gestating sows (air-dry basis, %).

Item	Content
Ingredients ^1^	
Yellow corn	65.0
Soybean meal	24.3
Soybean oil	2.5
Steam fish meal	3.0
Fine stone powder	1.2
Glucose	1.0
DL-Methionine	0.08
L-Threonine	0.07
L-Lysine·HCL	0.35
CaHPO4	1.0
NaCL	0.5
Vitamin–mineral premix1	1.0
Total	100.0
Nutrient levels ^2^	
ME, MJ/kg	13.4
Crude protein	17.0
Total P	0.66
Ca	0.85

^1^ The vitamin–mineral premix provided the following per kilogram of the basal diets: sweetening agent 200 mg, antioxidant 100 mg, vitamin D3 3000 IU, vitamin E 20 IU, vitamin K3 1.8 mg, vitamin A 6000 IU, riboflavin 6.0 mg, thiamine 2.0 mg, pyridoxine 4.0 mg, vitamin B12 0.02 mg, niacin 26.0 mg, pantothenic acid 18.0 mg, folic acid 3.2 mg, biotin 0.4 mg, Zn (as ZnSO_4_, H_2_O) 100 mg, Cu (as CuSO_4_, 5H_2_O) 20 mg, Mn (as MnSO_4_, H_2_O) 50 mg, Se (as Na_2_SeO_3_) 0.30 mg, I (as KI) 1.2 mg. ^2^ The nutrient levels were calculated values.

**Table 2:** Sequence of primers for quantitative real-time PCR.

Genes	Accession No.	Nucleotide Sequence of Primers (5′-3′)
ELOVL5	XM_021098832.1	F: TACCACCATGCCACTATGCT
		R: GACGTGGATGAAGCTGTTGA
FADS1	NM_001113041.1	F: GTCACTGCCTGGCTCATTCT
		R: AGGTGGTTCCACGTAGAGGT
FADS2	NM_001171750.1	F: ACGGCCTTCATCCTTGCTAC R: GTTGGCAGAGGCACCCTTTA
SREBP-1c	XM_021066226.1	F: GACCGGCTCTCCATAGACAA
		R: CCTCTGTCTCTCCTGCAACC
AKT	NP_001315268	F: TCAAGAACGACGGCACCTTCATC
		R: CGCCACGGAGAAGTTGTTGAGG
FAT/CD36	XM_021102279.1	F: CTGGTGCTGTCATTGGAGCAGT
		R: CTGTCTGTAAACTTCCGTGCCTGTT
PPAR-γ	XM_005669788.3	F: GTGGAGACCGCCCAGGTTTG
		R: GGGAGGACTCTGGGTGGTTCA
PC	NP_999234	F: CCGCAAGATGGGAGACAAGGT
		R: GGAAGCCGATGGTGTTGGAAGAA
PCK1	NP_999306	F: TCAGCACGACTCCAGCCTTCA
		R: GCTCAAGCAGTCTGGGCATTCT
G6PC	NM_001113445.1	F: AAGCCAAGCGAAGGTGTGAGC
		R: GGAACGGGAACCACTTGCTGAG
GLUT1	XM_021096908.1	F: GCAGGAGATGAAGGAGGAGAGC
		R: ACCAACAGCGACACGACAGT
GLUT2	XM_021092392.1	F: GCCCTGAAAGTCCTCGGTTCCT
		R: ACACGGCGTTGATGCCAGAGA
PEPCK-c	NP_001180252	F: AGTGGGATGGTGCAACTTGA
		R: CACATCACATCCACAGGGTG
PEPCK-m	NP_001180253	F: ATGGGCGGGTGCAACTTGA
		R: TCAGGTTGCCACAGGGTGG
HMGCR	NP_001245011	F: AAACCTGCTGCTGTAAACTGG
		R: GACCTCAACCATCGCTTCTG
GAPDH	NM_001206359.1	F: GTCTGGAGAAACCTGCCAAA
		R: CCCTGTTGCTGTAGCCAAAT
FABP2	NM_213979.1	F: TCCACCGCACGCTGGTCTAT
		R: CCAGTCCTCCTGCCTTCTCCAT
ICDH-γ	NM_001164007.1	F: TGTGGTTCCTGGTGAGAG
		R: CGAGATTGAGATGCCGTAG

**Table 3:** Impact of lecithin supplementation on sow reproductive performance.

Item	Treatment		*p*-Value
	CON	Lecithin	
Total born, n	13.42 ± 0.679	13.36 ± 0.801	0.960
Born alive, n	12.17 ± 0.747	12.82 ± 0.698	0.533
Stillbirth, n	0.42 ± 0.193	0.27 ± 0.141	0.559
Stillbirth rate, %	1.03 ± 0.679	1.97 ± 1.029	0.446
IUGR piglet, n	0.42 ± 0.193	0.09 ± 0.011	0.153
IUGR rate, %	2.19 ± 1.242	0.53 ± 0.035	0.248

Data are presented as mean ± SEM, n = 12.

**Table 4:** Impact of lecithin supplementation on piglet performance.

Item	Treatment		*p*-Value
	CON	Lecithin	
Litter birth weight, kg	23.41 ± 0.37	21.64 ± 0.60	0.227
Birth weight, kg	1.75 ± 0.192	1.74 ± 0.235	0.943
7 d Litter weight, kg	32.21 ± 0.68	30.65 ± 2.38	0.339
14 d Litter weight, kg	58.80 ± 1.24	59.85 ± 3.29	0.677
21 d Litter weight, kg	75.56 ± 1.85	77.14 ± 3.90	0.617
1–7 d average daily gain, g	140.15 ± 11.636	181.15 ± 7.925	0.011
7–14 d average daily gain, g	431.94 ± 24.834	454.54 ± 13.129	0.409
14–21 d average daily gain, g	236.95 ± 15.698	242.690 ± 12.011	0.408
1–21 d average daily gain, g	254.66 ± 14.693	261.72 ± 7.191	0.282

Data are presented as mean ± SEM, n = 12.

**Table 5:** The serum biochemical parameters of sows during pregnancy and lactation.

Item	Treatment		*p*-Value	Treatment		*p-*Value
	CON	Lecithin		CON	Lecithin	
TG, mmol/L	0.30 ± 0.07	0.18 ± 0.04	0.184	0.16 ± 0.03	0.12 ± 0.02	0.393
TC, mmol/L	1.76 ± 0.11	1.33 ± 0.10	0.018	1.76 ± 0.22	2.57 ± 0.32	0.065
LDL, mmol/L	0.98 ± 0.05	0.81 ± 0.10	0.116	0.79 ± 0.09	1.17 ± 0.17	0.072
HDL, mmol/L	0.77 ± 0.08	0.50 ± 0.04	0.019	0.98 ± 0.16	1.51 ± 0.20	0.066
GLU, mmol/L	4.92 ± 0.31	4.42 ± 0.59	0.428	2.60 ± 0.33	3.58 ± 0.36	0.071
CHE, U/L	484.62 ± 23.77	419.17 ± 42.76	0.179	408.33 ± 58.32	585.50 ± 48.20	0.041
LACT, mmol/L	3.12 ± 0.60	2.47 ± 0.48	0.433	5.07 ± 0.74	5.67 ± 0.52	0.055
LIPC, U/L	6.67 ± 1.09	8.77 ± 3.00	0.481	3.67 ± 0.55	6.28 ± 0.69	0.522
TBA, Umol/L	12.68 ± 2.98	20.90 ± 5.39	0.180	27.17 ± 4.16	37.35 ± 8.53	0.014

TG = total triglyceride; TC = total cholesterol; LDL = low-density lipoprotein; HDL = high-density lipoprotein; GLU = glucose; CHE = cholinesterase; LACT = D-lactic acid; LIPC = hepatic lipase; TBA = total bile acid. The contents of the subsequent forms are the same. Data are presented as mean ± SEM, n = 6.

**Table 6:** Serum biochemical parameters in umbilical cord and 21-day-old suckling piglet.

Item	Umbilical Cord		*p*-Value	Serum of Suckling Pig		*p-*Value
	CON	Lecithin		CON	Lecithin	
TG, mmol/L	0.26 ± 0.03	0.33 ± 0.05	0.205	0.89 ± 0.12	0.89 ± 0.07	0.981
TC, mmol/L	1.04 ± 0.15	1.39 ± 0.14	0.208	6.44 ± 0.43	5.85 ± 0.52	0.399
LDL, mmol/L	0.53 ± 0.09	0.74 ± 0.10	0.198	4.73 ± 0.45	3.83 ± 0.53	0.225
HDL, mmol/L	0.37 ± 0.07	0.48 ± 0.05	0.416	2.22 ± 0.20	2.48 ± 0.14	0.303
GLU, mmol/L	0.21 ± 0.04	0.23 ± 0.10	0.911	5.60 ± 0.45	7.00 ± 0.43	0.050
CHE, U/L	228.50 ± 30.66	308.00 ± 52.72	0.264	597.67 ± 29.89	614.17 ± 27.44	0.693
LACT, mmol/L	20.13 ± 0.95	18.05 ± 3.22	0.624	15.53 ± 2.22	11.77 ± 1.91	0.228
LIPC, U/L	2.38 ± 0.24	4.68 ± 1.17	0.065	5.07 ± 0.53	5.12 ± 0.27	0.935
TBA, Umol/L	4.88 ± 0.96	4.50 ± 1.12	0.804	29.15 ± 5.56	33.23 ± 2.45	0.517

TG = total triglyceride; TC = total cholesterol; LDL = low-density lipoprotein; HDL = high-density lipoprotein; GLU = glucose; CHE = cholinesterase; LACT = D-lactic acid; LIPC = lipase; TBA = total bile acid. Data are presented as mean ± SEM, n = 6.

**Table 7:** Fatty acids of serum and liver in the 21 d suckling piglets.

Item	Plasma of Piglets		*p*-Value	Liver of Piglets		*p-*Value
	CON	Lecithin		CON	Lecithin	
C6:0	—	—	—	0.09 ± 0.01	0.08 ± 0.01	0.406
C12:0	—	—	—	0.09 ± 0.00	0.08 ± 0.01	0.150
C14:0	—	—	—	0.25 ± 0.03	0.22 ± 0.02	0.443
C15:0	—	—	—	0.03 ± .0.01	0.04 ± 0.01	0.422
C16:0	22.87 ± 1.20	21.12 ± 0.57	0.214	15.73 ± 0.31	15.80 ± 0.33	0.869
C16:1	1.82 ± 0.17	1.88 ± 0.10	0.773	1.85 ± 0.27	1.81 ± 0.17	0.899
C17:0	0.16 ± 0.03	0.14 ± 0.01	0.496	0.17 ± 0.01	0.17 ± 0.01	0.868
C18:0	17.80 ± 1.58	16.41 ± 0.51	0.422	24.93 ± 0.92	25.34 ± 0.44	0.689
C18:1n9t	—	—		0.08 ± 0.01	0.07 ± 0.00	0.050
C18:1n9c	14.19 ± 1.95	11.59 ± 1.62	0.328	9.61 ± 0.42	8.76 ± 0.31	0.133
C18:2n6c	18.37 ± 2.13	16.92 ± 0.44	0.520	16.53 ± 0.58	16.00 ± 0.35	0.455
C18:3n3	0.40 ± 0.03	0.41 ± 0.01	0.659	0.15 ± 0.01	0.15 ± 0.01	0.901
C18:3n6	—	—	—	0.28 ± 0.05	0.26 ± 0.03	0.676
C20:0	—	—	—	0.06 ± 0.01	0.05 ± 0.01	0.072
C20:1	—	—	—	0.12 ± 0.01	0.11 ± 0.01	0.135
C20:2	0.37 ± 0.04	0.37 ± 0.02	0.906	0.48 ± 0.03	0.43 ± 0.02	0.232
C20:3n6	1.00 ± 0.08	0.85 ± 0.03	0.113	0.97 ± 0.06	0.79 ± 0.01	0.026
C20:4n6	23.11 ± 1.68	24.98 ± 1.30	0.397	24.00 ± 0.95	25.26 ± 0.26	0.229
C24:0	—	—	—	0.38 ± 0.02	0.28 ± 0.02	0.007
C20:5n3	—	—	—	0.03 ± 0.01	0.02 ± 0.01	0.679
C22:6n3	4.63 ± 0.32	4.68 ± 0.42	0.923	4.04 ± 0.40	4.23 ± 0.34	0.726
ΣSFA	40.75 ± 2.75	37.63 ± 0.39	0.288	41.72 ± 0.73	42.06 ± 0.41	0.688
ΣMUFA	15.71 ± 1.86	13.46 ± 1.56	0.377	11.67 ± 0.62	10.75 ± 0.37	0.231
ΣPUFA	42.84 ± 3.97	48.25 ± 1.69	0.238	46.47 ± 0.84	47.15 ± 0.31	0.467

Date is presented as mean ± SEM, n = 6. SFA = saturated fatty acids; MUFA = monounsaturated fatty acids; PUFA = polyunsaturated fatty acids. Total SFA includes C6:0, C12:0, C14:0, C15:0, C16:0, C17:0, C18:0, C20:0 and C24:0; total MUFA includes C16:1 C18:1n9c, C18:1n9c and C20:1; total PUFA includes C18:2n6c, C18:3n6, C18:3n3, C20:2, C20:3n6, C20:4n6, C20:5n3 and C22:6n3.

## Data Availability

Data may be provided upon request to the corresponding author (xie.chunyan@foxmail.com).
